# Bruton Tyrosine Kinase Inhibition in Combination With Bispecific Antibodies for Richter's Syndrome: A Report of Two Cases and Mini Review

**DOI:** 10.7759/cureus.104529

**Published:** 2026-03-02

**Authors:** Ben C Hane, Marc Hoffmann, Da Zhang, Saurav Chopra, Joseph Bennett

**Affiliations:** 1 Department of Hematologic Malignancies and Cellular Therapeutics, University of Kansas Medical Center, Kansas City, USA; 2 Department of Pathology and Laboratory Medicine, University of Kansas Health System, Kansas City, USA; 3 Department of Pathology and Laboratory Medicine, University of Kansas Medical Center, Kansas City, USA

**Keywords:** bispecific antibodies, bruton tyrosine kinase inhibitor, combination cancer immunotherapy, refractory diffuse large b-cell lymphoma, richter's syndrome

## Abstract

Chronic lymphocytic leukemia (CLL) is the most common adult leukemia in the United States. One feared complication of CLL is Richter’s syndrome (RS), where the leukemia transforms into a more aggressive lymphoma, most commonly diffuse large B-cell lymphoma (DLBCL). The current standard of care aims to achieve remission with chemoimmunotherapy; however, responses are limited, prompting investigations into novel treatment options. While both pirtobrutinib and epcoritamab have demonstrated efficacy as monotherapies, their combination may represent a promising strategy with potential synergistic effects. Although it remains unclear whether the immunomodulatory effects observed with covalent Bruton’s tyrosine kinase inhibitors (BTKis) will also occur with more selective, noncovalent agents such as pirtobrutinib, recent studies suggest that coadministration may potentiate bispecific antibody-mediated cytotoxicity. We discuss two cases that illustrate a management strategy incorporating combination therapy with epcoritamab and pirtobrutinib for DLBCL-RS in the relapsed or refractory setting.

## Introduction

Chronic lymphocytic leukemia (CLL) is an indolent leukemia with an age-standardized five-year net survival of approximately 77% [1]. It results from the clonal proliferation of mature B lymphocytes [2]. Symptoms arise from lymphocytic infiltration of the lymph nodes, blood, and bone marrow, as well as from the loss of antigenic diversity. Richter's syndrome (RS) occurs when CLL transforms into a more aggressive lymphoma, most commonly diffuse large B-cell lymphoma (DLBCL) [3]. RS occurs in 2-10% of patients with CLL and is associated with a significantly worse prognosis [4].

The current standard of care for DLBCL-RS aims to achieve remission with chemoimmunotherapy regimes such as R-CHOP (rituximab, cyclophosphamide, doxorubicin, vincristine, and prednisone) followed by stem cell transplantation. However, responses to chemoimmunotherapy are limited, with overall response rates (ORR) of 67% for R-CHOP, and are further complicated by high rates of adverse events [5]. Additionally, many patients with DLBCL-RS are not candidates for stem cell transplantation due to advanced age, comorbidities, or disease status [5]. Recent data suggest promising single-agent activity of the noncovalent Bruton’s tyrosine kinase inhibitor (BTKi) pirtobrutinib (ORR: 50%, complete response (CR): 13%) [6] and the anti-CD3/CD20 bispecific monoclonal antibody epcoritamab (ORR: 50%, CR: 35%) [7]. Noncovalent BTKis offer several benefits over their covalent BTKi counterparts. One of the primary mechanisms of resistance to covalent BTKis is a point mutation in the C481 binding site on BTK.

Because noncovalent BTKi do not require C481 binding for inhibition, they remain active in the setting of this mutation. Noncovalent BTKi’s are also highly selective for BTK over other kinases, reducing the risk of off-target toxicities [8]. CAR-T data from CLL suggest that combining T-cell re-directing immunotherapy with BTK inhibition may have synergistic effects, either through direct stimulation of endogenous T-cells, creating healthier populations of T-cells to be used for the CAR-T construct, or activity against CLL. Combination therapy with ibrutinib and lisocabtagene maraleucel (liso-cel) offered higher ORR (86 vs. 43%) and CR (45 vs. 18%) compared to liso-cel alone in relapsed or refractory CLL patients. Given these data, it was hypothesized that combination therapy with pirtobrutinib and epcoritamab may offer improved efficacy and potential synergy [9]. We present two patients with DLBCL-RS who were not candidates for CAR T-cell therapy and were successfully treated with epcoritamab and pirtobrutinib.

## Case presentation

Case 1

A 73-year-old Caucasian male with no prior medical history presented in 2016 with asymptomatic leukocytosis and was monitored until January 2018, when his white blood cell count reached 125.37 x 10³/μL. Fluorescence in-situ hybridization (FISH) was negative for MYB, SEC63, ATM, TP53, RB1, and 13q deletions, trisomy 12, and CCND1/IGH fusion. He began bendamustine and rituximab, but it was discontinued after cycle 1, dose 1, due to prolonged cytopenia. He remained off treatment until August 2018, when a fever of unknown origin prompted a bone marrow biopsy (BMBx) showing CLL and sheets of large cells with features suggestive of large cell transformation. From September to December 2018, he received six cycles of R-CHOP. Repeat BMBx in January 2019 revealed 5-10% marrow involvement by CLL and no residual large cell lymphoma; however, PET-CT showed skeletal lesions confirmed as DLBCL by biopsy. Nivolumab monotherapy was initiated, and four months later, ibrutinib was added. Restaging PET-CT in April 2019 showed CR. He declined allogeneic stem cell transplantation and remained on ibrutinib and nivolumab until August 2023, when he discontinued both due to concerns of new-onset atrial fibrillation and recurrent infections.

**Figure 1:**
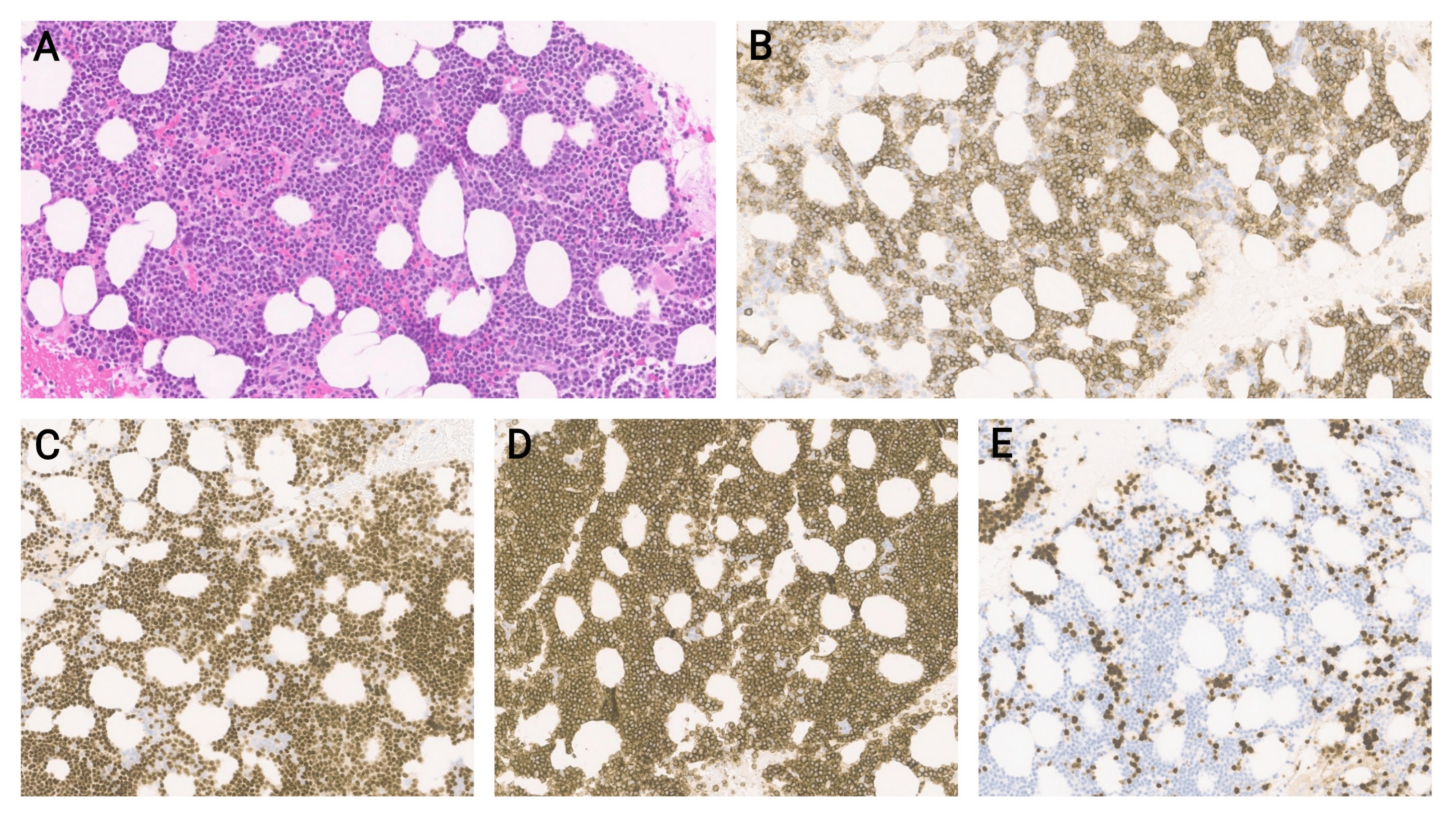
Bone marrow biopsy showing a hypercellular marrow with extensive involvement by CLL/SLL (approximately 70-80%), associated with decreased trilineage hematopoiesis and a low proliferation index No definitive morphologic evidence of large cell transformation was identified in the marrow A. H&E stain showing bone marrow with interstitial and nodular lymphoid infiltrates. B. CD20 immunohistochemistry highlighting extensive marrow involvement by CLL/SLL (100×). C. PAX5 immunohistochemistry highlighting extensive marrow involvement by CLL/SLL (100×). D. CD5 immunohistochemistry showing co-expression on neoplastic B cells, consistent with CLL/SLL (100×). E. Ki-67 immunohistochemistry demonstrating a low proliferation index in lymphoid infiltrate, and showing some staining in background bone marrow cells CLL: chronic lymphocytic leukemia; SLL: small lymphocytic lymphoma; H&E: hematoxylin and eosin

**Figure 2:**
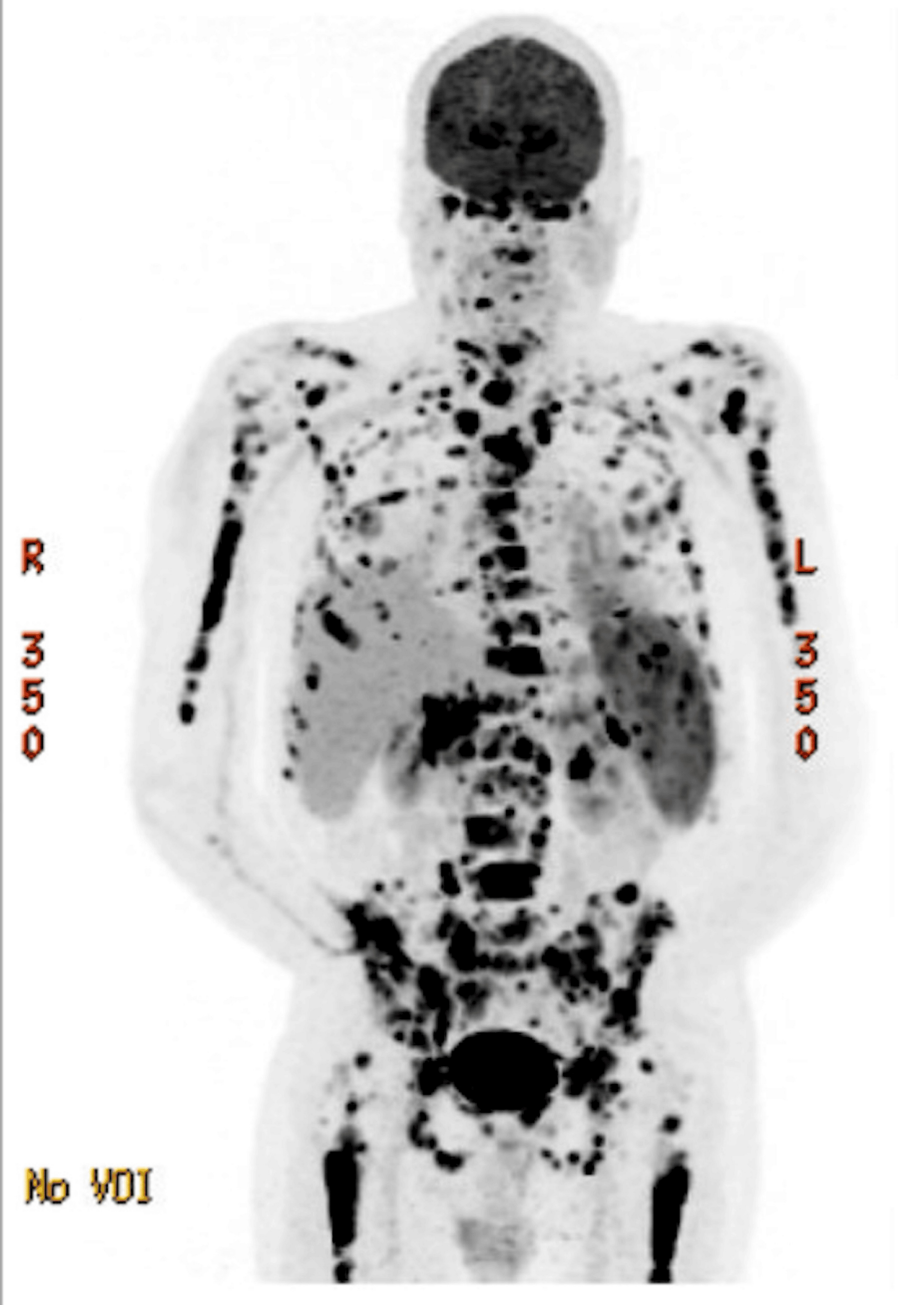
PET-CT findings before epcoritamab and pirtobrutinib combination therapy Findings are compatible with relapsed high-grade or transformed disease (5 PS = 5). Representative hypermetabolic right inguinal lymph node demonstrates a maximum SUV of 22.4 PET-CT: positron emission tomography-computed tomography; SUV: standard uptake variable

Restaging BMBx in February 2024 revealed 10% involvement with CLL with next-generation sequencing (NGS) negative for deleterious mutations. Active surveillance was pursued. In March 2025, he presented with lethargy, general weakness, and fever. BMBx showed CLL involving 70-80% of a hypercellular bone marrow (50-60% cellularity) with decreased trilineage hematopoiesis and less than 1% blasts (Figure 1). PET scan showed diffuse hypermetabolic lymphadenopathy with maximum standard uptake variable (SUV) of 22.4, splenomegaly with multifocal splenic hypermetabolism, and axial and appendicular skeletal lesions (Figure 2).

Right inguinal lymph node biopsy confirmed recurrent Richter’s transformation. NGS was again negative, and bone marrow biopsy FISH panel was negative for 6q, 11q, trisomy 12, TP53, or 13q cytogenetic abnormalities. The patient started 200 mg pirtobrutinib orally daily and a rapid ramp up of subcutaneous epcoritamab (0.16mg C1D1 -> 48 mg C1D5). Four days after the initial dose of epcoritamab, he developed grade 2 cytokine release syndrome (CRS) and grade 2 immune effector cells-associated neurotoxicity syndrome (ICANS), which resolved with tocilizumab and dexamethasone. 

**Figure 3:**
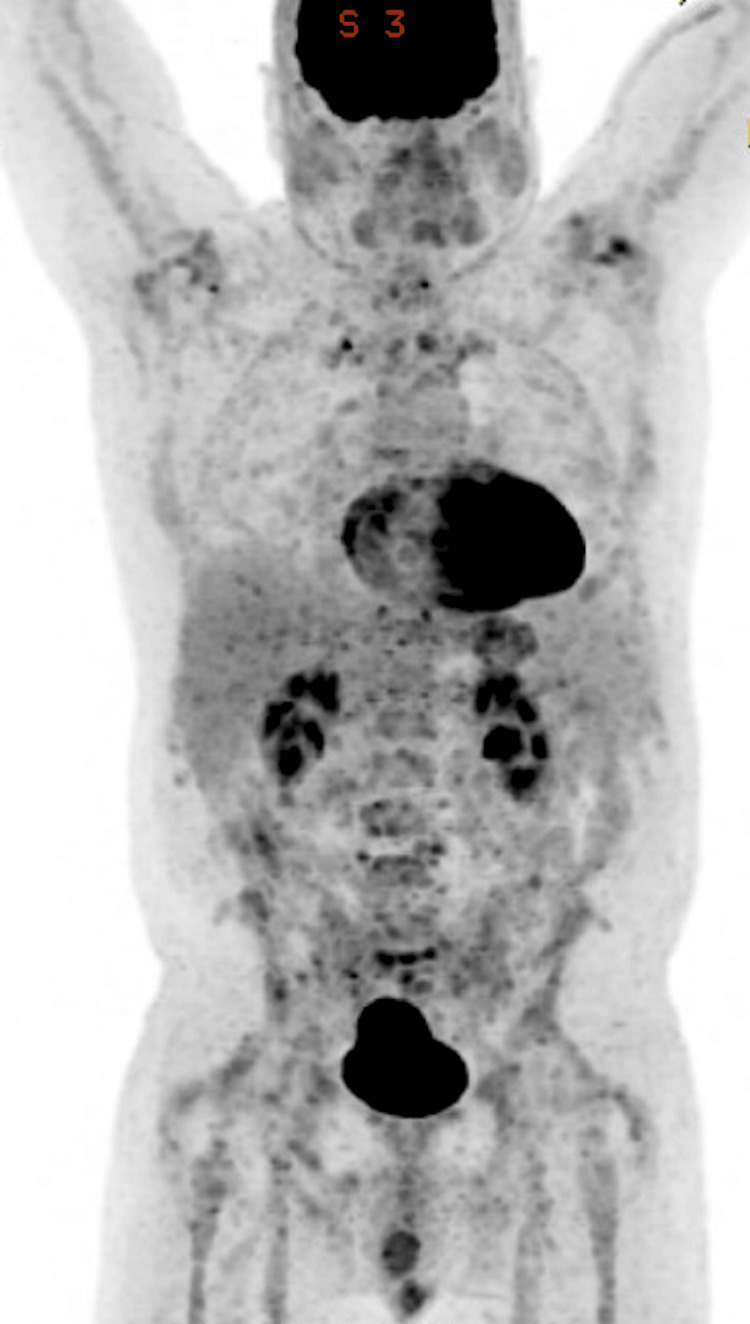
PET-CT findings after three cycles of epcoritamab and pirtobrutinib combination therapy Resolution of previous hypermetabolic lymphadenopathy and multifocal marrow and splenic lesions. Findings are compatible with complete response (5 PS = 1) PET-CT: positron emission tomography-computed tomography

After three months and three cycles of therapy, PET-CT and bone marrow biopsy showed CR with undetectable minimal residual disease by flow cytometry (uMRDx10-4). Figure 3 demonstrates the patient's response after three cycles of epcoritamab and pirtobrutinib. Nine months after starting epcoritamab/pirtobrutinib therapy, he remains in CR with no notable toxicities. Table 1 provides a summary of the patient characteristics and treatment course.

**Table 1:** Summary of patient characteristics and treatment course - case 1 CLL: chronic lymphocytic leukemia; FISH: fluorescence in-situ hybridization; NGS: next-generation sequencing; BMBx: bone marrow biopsy; PET-CT: positron emission tomography-computed tomography; DLBCL: diffuse large B-cell lymphoma; CR: complete response

Summary	
Baseline characteristics	
Age at presentation	73 years
Sex	Male
Ethnicity	Caucasian
Initial diagnosis	CLL, 2016
FISH (2018)	Negative for MYB, SEC63, ATM, TP53, RB1, 13q deletion, trisomy 12, CCND1/IGH
NGS (Feb 2024)	Negative for deleterious mutations
Prior therapies and timeline	
Line 1 (Jan 2018)	Bendamustine + rituximab; discontinued after cycle 1, day 1 due to prolonged cytopenia
Off treatment	January 2018 - August 2018
Bone marrow biopsy (Aug 2018)	CLL with features of large cell transformation (Richter's)
Line 2 (Sep-Dec 2018)	R-CHOP × 6 cycles
Response to R-CHOP	BMBx (Jan 2019): 5-10% CLL involvement, no residual large cell lymphoma; PET-CT: skeletal DLBCL lesions
Line 3 (early 2019)	Nivolumab monotherapy, then ibrutinib added ~4 months later
Response to nivolumab + ibrutinib	CR - PET-CT April 2019
Duration of response	~4 years (April 2019 - August 2023)
Reason for discontinuation	New onset atrial fibrillation and recurrent infections (August 2023)
Surveillance period	February 2024: BMBx 10% CLL, NGS negative → active surveillance
Current therapy	
Relapse presentation (Mar 2025)	Lethargy, weakness, fever; BMBx 70-80% CLL in hypercellular marrow; PET: diffuse hypermetabolic lymphadenopathy (max SUV 22.4), splenomegaly, skeletal lesions
Current regimen	Pirtobrutinib 200 mg orally daily + epcoritamab (rapid ramp-up: 0.16 mg C1D1 → 48 mg C1D5)
Notable toxicities	Grade 2 CRS and Grade 2 ICANS (4 days post-epcoritamab); resolved with tocilizumab and dexamethasone
Response (3 months/3 cycles)	CR with uMRD by flow cytometry (uMRD × 10⁻⁴)
Current status	CR at 9 months; no notable toxicities

Case 2

A 60-year-old Caucasian male with a past medical history of methamphetamine abuse was diagnosed with CLL in 2016 after presenting with bilateral axillary lymphadenopathy and malaise. Peripheral blood flow analysis demonstrated lymphocytes comprising 87% of total events. Flow cytometry of B cells was positive for CD5, CD19, CD20, CD23, and CD200. They were negative for CD10, CD38, FMC-7, and ZAP 70. FISH of peripheral blood showed 11q deletion, 13q deletion, and 17p deletion. CT demonstrated bulky axillary and mild mediastinal lymphadenopathy, bulky retroperitoneal and pelvic lymphadenopathy, moderate splenomegaly, and left hydronephrosis likely secondary to the mass effect of the distal ureter by lymphadenopathy. Axillary lymph node biopsy showed mature lymphocytes forming pseudo-germinal centers with neoplastic cells positive for CD20, CD5, and BCL-2 and negative for CD3, CD10, BCL-6, and cyclin D1.

The patient was started on ibrutinib 420 mg orally daily with good response; however, after one year, he developed rapid lymphadenopathy. Biopsy demonstrated DLBCL with immunostaining positive for BCL2 and BCL6 and negative for cMYC. BMBx showed hypercellular marrow (90-95% lymphocytes), and PET-CT showed diffuse lymphadenopathy with a maximum SUV of 14. Ibrutinib was discontinued, and he received six cycles of DA-R-EPOCH and achieved a partial response. He was not a transplant candidate due to substance use, and hence, ibrutinib maintenance was resumed.

After one year, the patient developed worsening right axillary lymphadenopathy, prompting a biopsy, which demonstrated Richter’s transformation. In April 2019, he received salvage chemotherapy with rituximab + methotrexate + cytarabine (part B hyper-CVAD) while continuing ibrutinib. PET scan after two cycles showed response with only mild residual activity at the right axillary lymph node surgical site and low-grade increased uptake in the abdominal lymph nodes. He continued ibrutinib due to ongoing ineligibility for transplant, with a plan for staging evaluation in case of clinical progression. In February 2020, progressive lymphadenopathy prompted a biopsy confirming DLBCL. He received a total of four cycles of rituximab, gemcitabine, and oxaliplatin, with ibrutinib continued throughout treatment, but he was lost to follow-up. He returned in August 2020, asymptomatic, to discuss his plan of care. A restaging PET-CT demonstrated CR five months after his last cycle of R-Gem-Ox. Nivolumab was initiated in combination with ibrutinib to maintain remission.

**Figure 4:**
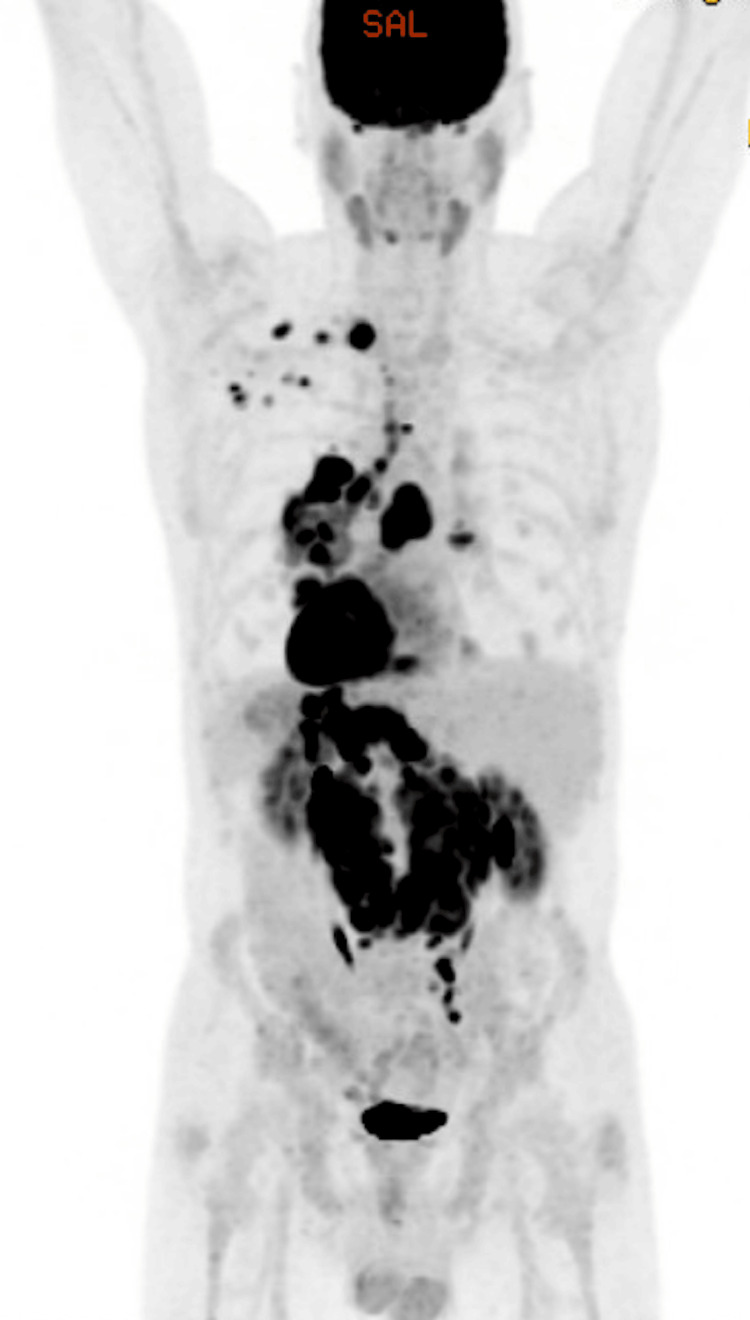
PET-CT findings before epcoritamab and pirtobrutinib combination therapy FDG-avid left supraclavicular, left axillary, mediastinal, bilateral hilar, gastrohepatic, and retroperitoneal lymph nodes are noted. The most FDG-avid is a left hilar lymph node with a max SUV of 14.3 PET-CT: positron emission tomography-computed tomography; FDG: fluorodeoxyglucose; SUV: standard uptake variable

**Figure 5:**
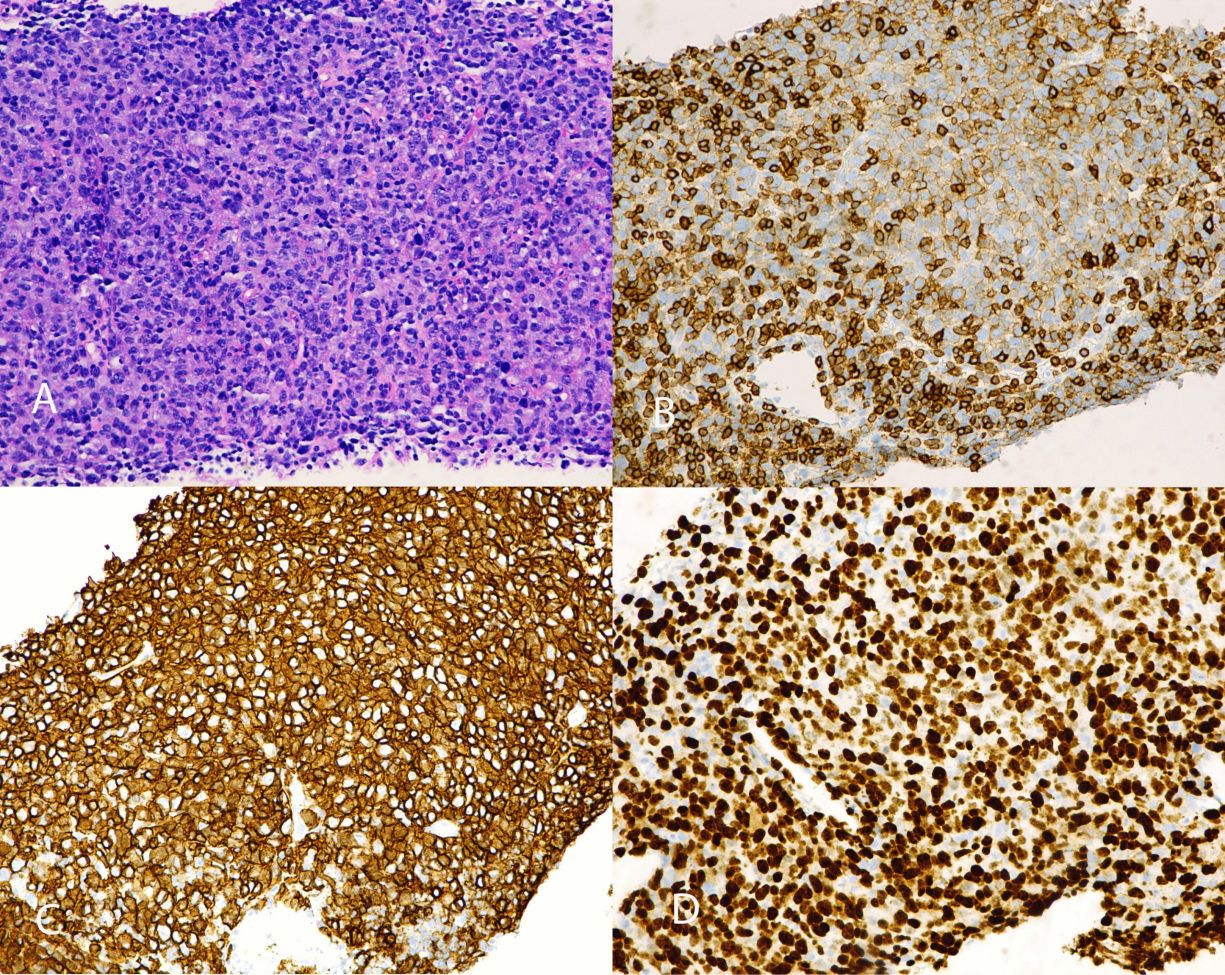
Retroperitoneal mass, needle core biopsy (A, H&E stain, 20X) The biopsy shows a diffuse lymphoid infiltrate composed of large, atypical cells with moderate nuclear size variation and irregular nuclear contours. Immunohistochemical studies demonstrate that these large atypical cells are positive for CD5 (B; weak staining in large cells, 20x), CD20 (C. 20x), and Ki‑67 with a high proliferation index (D; ~80%, 20x). Additional immunohistochemical stains (not shown) reveal that the neoplastic cells are positive for CD30 (∼85%), BCL2, CMYC, and MUM1, negative for CD3, CD10, and cyclin D1, and show borderline positivity for BCL6 (∼30%) H&E: hematoxylin and eosin

**Figure 6:**
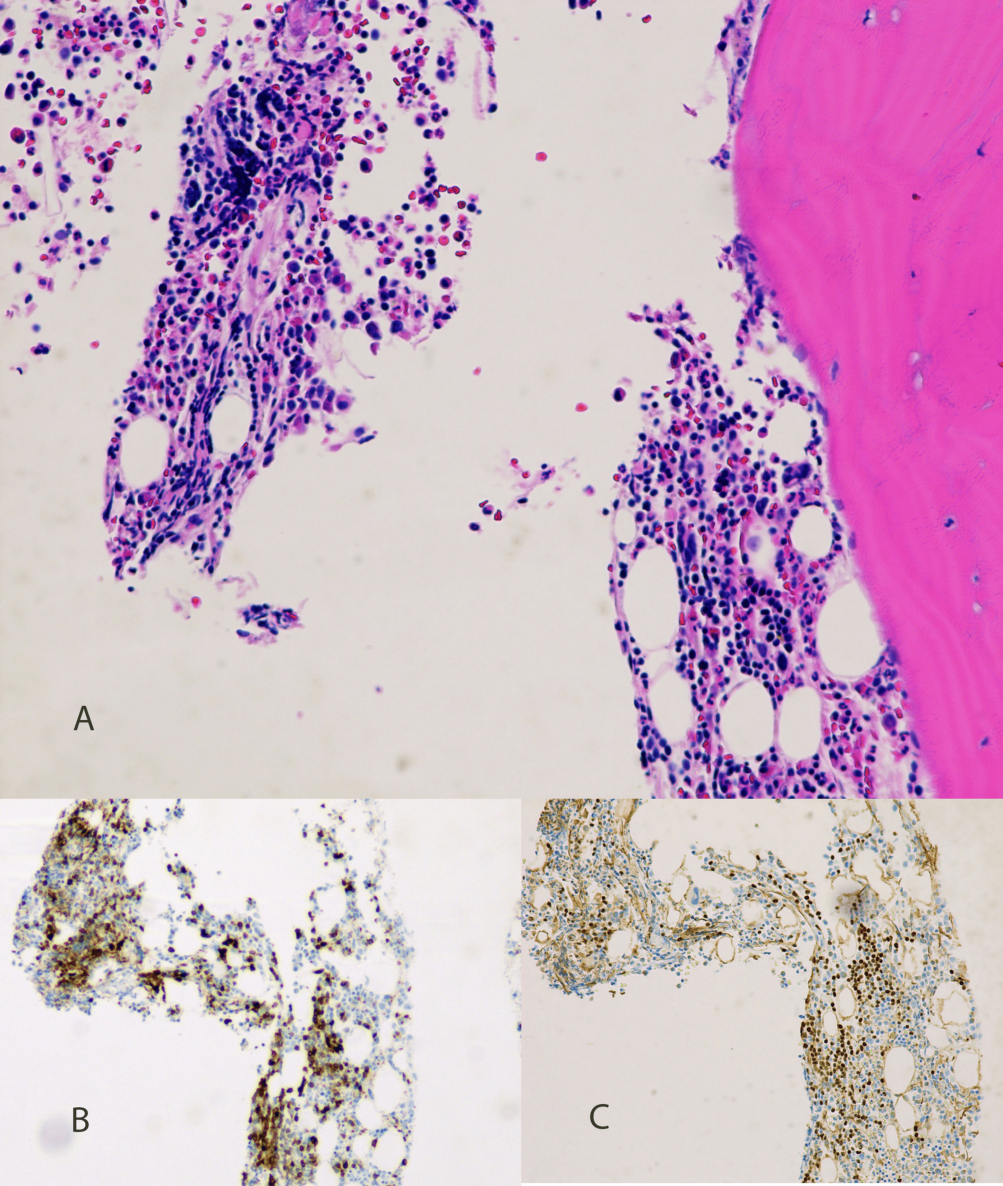
Bone marrow biopsy Bone marrow shows a normocellular marrow with trilineage hematopoiesis and rare, scattered small lymphocytes that are difficult to characterize without immunohistochemical stains (A, H&E, 20X). Immunohistochemical stains for CD5 (B, 20X) and PAX5 (C, 20X) highlight scattered B lymphocytes with aberrant co‑expression of CD5, a T‑cell–associated marker, comprising less than 5% of the marrow cellularity H&E: hematoxylin and eosin

The patient remained in CR for four years, receiving 21 cycles of nivolumab; however, in June of 2024, an abdominal discomfort prompted a PET-CT, which showed multifocal lymphadenopathy with a maximum SUV of 14.3 (Figure 4), a lymph node biopsy showing DLBCL (Figure 5), and BMBx showing normocellular marrow with less than 5% involvement by CLL (Figure 6). Three weeks later, he returned to the emergency department for constipation, nausea, and night sweats attributed to relapse of his CLL and DLBCL.

**Figure 7:**
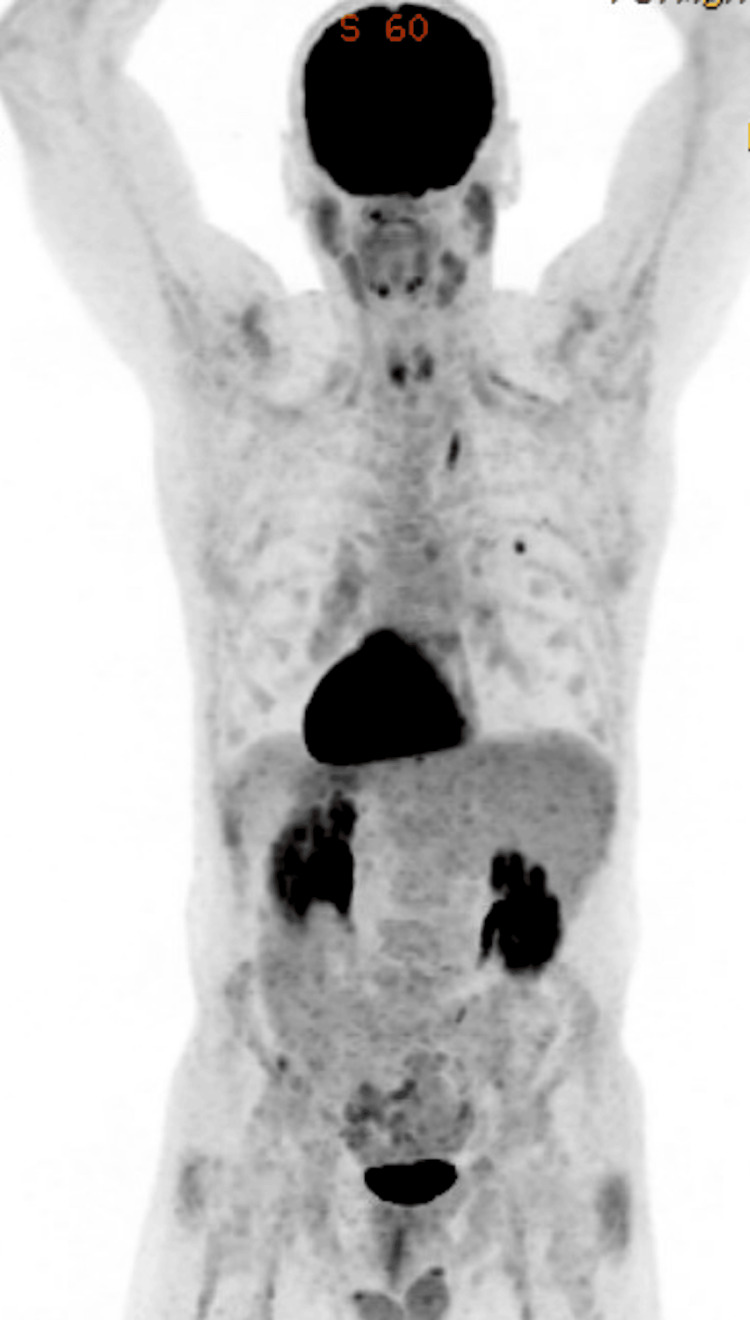
PET-CT findings after six cycles of epcoritamab and pirtobrutinib combination therapy Resolution of previously hypermetabolic left supra-and lymphadenopathy. Resolution of hypermetabolic mediastinal lymph nodes. Diminished mild peripheral uptake in the cystic left hilar node, which has decreased in size, with a maximum SUV of 2.9. Lesions are compatible with a favorable response to therapy (5PS = 3) PET-CT: positron emission tomography-computed tomography; SUV: standard uptake variable

During this admission, nivolumab was discontinued, ibrutinib held, and epcoritamab started with rapid ramp-up (0.16 mg C1D1 -> 48 mg C1D7). C1D3 was held because of grade 2 CRS, which resolved with tocilizumab. Due to rapidly progressive lymphocytosis, ibrutinib therapy was resumed during cycle one, then, after one month, was replaced with pirtobrutinib 200 mg daily in July 2024. CT scan one month after initiation of epcoritamab and pirtobrutinib combination therapy showed near resolution of all abdominopelvic lymphadenopathy. PET scan in December 2024, five months after starting combination therapy, showed CR with lymph nodes with a maximum SUV of 2.9. Figure 4 demonstrates the PET-CT scan before the combination therapy. Figure 7 demonstrates the PET-CT scan after combination therapy. The patient has remained in CR on epcoritamab and pirtobrutinib combination therapy for 12 months, has been clinically asymptomatic, and has had no serious adverse effects. Table 2 provides a summary of the patient characteristics and treatment course.

**Table 2:** Summary of patient characteristics and treatment course - case 2 CLL: chronic lymphocytic leukemia; FISH: fluorescence in-situ hybridization; NGS: next-generation sequencing; DLBCL: diffuse large B-cell lymphoma; BMBx: bone marrow biopsy; CR: complete response; PET-CT: positron emission tomography-computed tomography

Summary	
Baseline demographics	
Age at diagnosis	60 years
Sex	Male
Ethnicity	Caucasian
Initial diagnosis	CLL, 2016
Flow cytometry (2018)	Positive: CD5, CD19, CD20, CD23, CD200; negative: CD10, CD38, FMC-7, ZAP-70
FISH (2018)	11q deletion, 13q deletion, 17p deletion
Lymph node biopsy	Mature lymphocytes with pseudo-germinal centers; positive CD20, CD5, BCL-2; negative CD3, CD10, BCL-6, cyclin D1
Prior therapies and timeline	
Line 1 (2016)	Ibrutinib 420 mg orally daily - good initial response
BMBx (2017)	After ~1 year on Ibrutinib: DLBCL confirmed by biopsy (BCL2+, BCL6+, cMYC−); BMBx 90–95% lymphocytes
Line 2 (~2017–2018)	DA-R-EPOCH × 6 cycles - partial response; ibrutinib maintenance resumed (ineligible for transplant due to substance use)
Line 3 (April 2019)	Rituximab + methotrexate + cytarabine (Hyper-CVAD part B) + ibrutinib continued; PET after 2 cycles showed response with mild residual activity
Maintenance post-line 3	Ibrutinib continued; ongoing transplant ineligibility; plan for restaging if clinical progression
Line 4 (2020)	R-gemcitabine-oxaliplatin (R-Gem-Ox) × 4 cycles + ibrutinib continued; lost to follow-up during treatment
Response to R-Gem-Ox	CR - PET-CT confirmed ~5 months after last cycle (August 2020)
Maintenance (Aug 2020)	Nivolumab added to ibrutinib to maintain remission
Duration of CR	~4 years (August 2020 – June 2024); 21 cycles of Nivolumab completed
Richter's transformation relapse and current therapy	
Relapse presentation (June 2024)	Abdominal discomfort; PET: multifocal lymphadenopathy, max of SUV 14.3; lymph node biopsy: DLBCL; BMBx: normocellular marrow, <5% CLL involvement
Current regimen (July 2024)	Pirtobrutinib 200 mg orally daily + epcoritamab (rapid ramp-up: 0.16 mg C1D1 → 48 mg C1D5)
Early response (1 month)	CT: near resolution of all abdominopelvic lymphadenopathy
Response (5 months - Dec 2024)	CR - PET-CT: lymph nodes, max SUV of 2.9
Current status	CR at 12 months on epcoritamab + pirtobrutinib; clinically asymptomatic; no serious adverse effects

## Discussion

Pirtobrutinib has proven efficacy as a monotherapy for DLBCL-RS, with a reported ORR of 50%, and 13% of patients achieving CR. In patients who previously experienced disease progression on covalent BTKi, the ORR was 42.0%, suggesting a role for pirtobrutinib in relapsed/refractory DLBCL-RS. However, the durability of responses is limited, with a median duration of response of 7.4 months. Pirtobrutinib demonstrated a favorable safety profile, with no patients discontinuing treatment due to adverse events [6]. Epcoritamab has also proven to be effective as a monotherapy in DLBCL-RS, with ORR and CR rates of 50% and 35%, respectively. At nine months, 53% of patients who achieved a CR remained in CR. No patients discontinued treatment due to adverse events [7].

While pirtobrutinib and epcoritamab have been effective as monotherapies in treating DLBCL-RS, it has been theorized that their combination may produce superior outcomes. This hypothesis is based on studies showing that ibrutinib has immunomodulatory effects that enhance the cytotoxicity of bispecific antibodies against CLL cells. These effects include downregulation of CTLA-4, an immunosuppressor, on CLL cells, increased T cell counts, and improved synapse formation between T cells and CLL cells [10]. The extent to which these immunomodulatory effects are attributable to ibrutinib’s BTK inhibition versus off-target kinase inhibition has not been fully elucidated [11-13]. It is not yet known whether pirtobrutinib, which has significantly fewer off-target effects than ibrutinib, will produce similar immunomodulatory effects.

Although pirtobrutinib may replicate only some of the immunomodulatory effects of Ibrutinib, it would likely still result in enhanced epcoritamab-mediated killing of CLL cells. A recent study showed that “epcoritamab induced a higher degree of T-cell activation, proliferation, and expression of cytotoxic effectors in T cells” in cultures cotreated with acalabrutinib [14]. This study demonstrated that BTKi therapy increases effector-to-target ratios, which positively correlated with enhanced epcoritamab-induced CLL cell lysis, providing a mechanistic basis for the synergy between BTKis and bispecific antibodies [14].

## Conclusions

Although pirtobrutinib and epcoritamab have shown efficacy as monotherapies, their combination represents a promising strategy with the potential for synergistic activity. These cases demonstrate that combination therapy with pirtobrutinib and epcoritamab resulted in a longer duration of CR than the median duration of response observed in monotherapy trials of either agent. Because CLL is predominantly a disease of older adults, a chemotherapy-sparing regimen using novel agents is an approach that merits further evaluation. However, it remains unclear whether the immunomodulatory effects observed with covalent BTK inhibitors will also occur with more selective, noncovalent BTK inhibitors such as pirtobrutinib. Future studies should evaluate the in vitro impact of pirtobrutinib coadministration on bispecific antibody-mediated cytotoxicity against CLL cells. Although limited to two patients, these case reports suggest a rapid and durable response to the epcoritamab and pirtobrutinib combination in DLBCL-RS and may offer a clinically meaningful treatment option for patients with relapsed or refractory disease. Larger prospective studies are required to validate these findings and to further define the safety and efficacy profile of this combination.
